# Notch1 Modulation of Cellular Calcium Regulates Mitochondrial Metabolism and Anti-Apoptotic Activity in T-Regulatory Cells

**DOI:** 10.3389/fimmu.2022.832159

**Published:** 2022-02-10

**Authors:** Neetu Saini, Sowmya Lakshminarayanan, Priyanka Kundu, Apurva Sarin

**Affiliations:** ^1^ Regulation of Cell Fate, Institute for Stem Cell Science and Regenerative Medicine (inStem), Bengaluru, India; ^2^ Department of Biology, Manipal Academy of Higher Education, Manipal, India; ^3^ National Centre for Biological Science, TATA Institute of Fundamental Research (TIFR), Bengaluru, India

**Keywords:** NOTCH1, calcium, apoptosis, mitochondria, oxphos, Grp75, Tregs, mammalian cells

## Abstract

As the major hub of metabolic activity and an organelle sequestering pro-apoptogenic intermediates, mitochondria lie at the crossroads of cellular decisions of death and survival. Intracellular calcium is a key regulator of these outcomes with rapid, uncontrolled uptake into mitochondria, activating pro-apoptotic cascades that trigger cell death. Here, we show that calcium uptake and mitochondrial metabolism in murine T-regulatory cells (Tregs) is tuned by Notch1 activity. Based on analysis of Tregs and the HEK cell line, we present evidence that modulation of cellular calcium dynamics underpins Notch1 regulation of mitochondrial homeostasis and consequently anti-apoptotic activity. Targeted siRNA-mediated ablations reveal dependency on molecules controlling calcium release from the endoplasmic reticulum (ER) and the chaperone, glucose-regulated protein 75 (Grp75), the associated protein Voltage Dependent Anion Channel (VDAC)1 and the Mitochondrial Calcium Uniporter (MCU), which together facilitate ER calcium transfer and uptake into the mitochondria. Endogenous Notch1 is detected in immune-complexes with Grp75 and VDAC1. Deficits in mitochondrial oxidative and survival in Notch1 deficient Tregs, were corrected by the expression of recombinant Notch1 intracellular domain, and in part by recombinant Grp75. Thus, the modulation of calcium dynamics and consequently mitochondrial metabolism underlies Treg survival in conditions of nutrient stress. This work positions a key role for Notch1 activity in these outcomes.

## Introduction

Cell survival depends on the availability of nutrients in the immediate environment and this can be a particular challenge in the immune system, where the cells are mobile and must move between regions of plentiful nutrients and sites where these might be limited (1, 2). The differentiation and survival of T-cell subsets is exquisitely dependent on cytokines, which control nutrient uptake in these cells and are thought to regulate cellular responses to environmental stress (3–5). Hence, the T-cell lineage offers a rich system to interrogate molecular mechanisms that regulate well-documented differences in survival outcomes in T-cell subsets.

In earlier work, we showed that Notch1 activity in Foxp3^+^ Tregs in the mammalian immune system, protects mitochondria from damage and promotes survival in cytokine (nutrient)-deprived culture conditions (5). Recapitulating these observations in mammalian cell lines, ligand-activated Notch1 intracellular (NIC)1 domain activity when enforced from the cytoplasm, conferred protection from apoptotic stimuli, including those targeting mitochondrial integrity (6–8). Notch signaling is a conserved pathway, initiated by interactions with ligand, and culminating in the release of the signaling active intermediate – NIC1 - from its full-length membrane-tethered precursor (9–12). While Notch signaling predominantly activates transcription, there is increasing evidence of non-transcriptional or non-canonical outcomes of this pathway (13–17). In many contexts, this is linked to NIC1 activity from different subcellular locations (13, 15, 18, 19).

Calcium is a well-known second messenger implicated in diverse signaling processes (20–24). While mitochondrial health is critically controlled by calcium, mitochondria reciprocally regulate the intracellular distribution of calcium. Thus, apoptotic signaling frequently correlates with changes in the levels and distribution of cellular calcium pools (24–27). Mitochondria are dynamic organelles whose shape and function respond to different physiological conditions by fusion, fission or biogenesis (27–30). The chaperone glucose related protein 75(Grp75) and voltage dependent anion channel (VDAC)1 are conduits for mitochondrial uptake of calcium, released from the major store of cellular calcium, the endoplasmic reticulum (ER) (31–33). VDAC1 interacts with Inositol 1,4,5-trisphosphate receptor(IP3R)3 on the ER *via* Grp75, at sites where mitochondria and ER are in close proximity, which results in localized elevations in calcium, facilitating uptake by mitochondria (33, 34). Mitochondria play many roles in cellular calcium homeostasis, including, calcium sequestration, activation of calcium dependent dehydrogenases, or, the activation of apoptotic pathways, which are coordinated by mitochondria, to name a few (27, 35, 36). The basal entry of calcium into mitochondria is required for diverse metabolic processes (37, 38). Controlled calcium uptake in mitochondria increases ATP production by activating calcium dependent dehydrogenases including pyruvate dehydrogenase (PDH), α-ketoglutarate dehydrogenase (α-KGDH), and isocitrate dehydrogenase (IDH) (39, 40). Many apoptotic stimuli cause calcium release from the ER to activate apoptotic cascades (41). Rapid overloading of calcium in mitochondria from the cytoplasm facilitates the opening of permeability transition pore followed by a loss of mitochondrial transmembrane potential (MTP) and release of cytochrome c and other pro-apoptogenic intermediates, which are usually sequestered in mitochondria (27). Bcl2 family anti-apoptotic proteins reduce free calcium levels in the ER (42, 43). Conversely, Bcl2 may increase uptake of calcium in mitochondria and compromised mitochondrial membrane potential has been shown to abrogate Bcl2 activity (44, 45).

It is increasingly appreciated that metabolic reprogramming is key to the differentiation of T-cell subsets (46, 47). In mammals, the functional maturation of T-cells in response to antigen, is a defining event in the adaptive immune response (48). The differentiation of T-cell subsets, is linked to programs initiated during immune activation, which in turn, are dependent on mitochondrial activity (49). Natural Tregs – where we have demonstrated a role for Notch1 activity - depend on fatty acid oxidation for function, although glycolysis is important in the initial responses to antigen. Cross-talk with the Notch1 pathway has been demonstrated with metabolic mediators and pathways that play critical roles in specialized T-cell subsets, especially Tregs (50–52). Here we explore the role of Notch1 in the regulation of metabolic (mitochondrial) activity vis-à-vis organellar calcium homeostasis, and IL-2 independent Treg survival.

## Materials And Methods

### Mice

The Notch1^lox/lox^ (Notch1^+/+^) and Cd4-Cre::Notch1^lox/lox^ (Notch1^-/-^) strains were a gift from Freddy Radtke (École Polytechnique Federale de Lausanne (EPFL), Switzerland) (53). C57BL/6J was obtained from the Jackson Laboratory. Tregs were isolated from spleens of 8-12 weeks old mice. Two spleens were pooled for the isolation of 2 million Tregs. Breeding colonies were maintained in-house in controlled temperature and light environments, in high barrier conditions, and in controlled systems (individually ventilated cages). Colonies were routinely monitored for the full pathogen panel recommended by the Federation of Laboratory Animal Science Associations. All experimental protocols were approved by the Institutional Animal Ethics Committee (INS-IAE-2019/07(R1)) and complied with the norms of the Committee for the Purpose of Control and Supervision of Experiments on Animals, Government of India.

### Cells

The HEK293T (HEK) cell line was from American Type Culture Collection (ATCC) (Manassas, VA, USA) and maintained in DMEM-CM containing Dulbecco’s Modified Eagle’s Medium (DMEM) (GIBCO, Life Technologies, Carlsbad, CA, USA) supplemented with 0.1% penicillin/streptomycin and 10% heat-inactivated fetal bovine serum (Scientific Hyclone TM, Waltham, MA, USA) at 37°C with 5% CO_2_. T cells were cultured in RPMI 1640 (GIBCO, Life Technologies, Carlsbad, CA, USA) supplemented with 0.1% penicillin/streptomycin with5% heat-inactivated fetal bovine serum (RPMI-complete medium) at 37°C with 5% CO_2_. Mycoplasma contamination in the cultures was routinely tested using the MycoAlertTM Mycoplasma Detection Kit (Lonza, Basel, Switzerland).

### Chemical and Antibodies

Thapsigargin (TG, T9033), flurorcarbonyl cyanide phenylhydrazone (FCCP, C2920), oligomycin (75351), rotenone (R8875), antimycin A (A8674), MKT-077 (M5549), 2-deoxy glucose (D8375), and histopaque (10831) were purchased from Sigma-Aldrich (St. Louis, MO, USA). γ-Secretase Inhibitor X (GSI-X, 565771), Ru360 (557440), and puromycin (508838) were from Calbiochem-Merck Millipore (Darmstadt, Germany). Xestospongin C (1280) was purchased from Tocris (Abingdon, UK). Dharmafect-1 and siRNA to scrambled control (D-0018010), MCU (L-015519), VDAC1 (L-019764), Grp75 (L-004750), IP3R3 (L-006209) and MFN2 (L-012961) were from Dharmacon (Lafayette, CO, USA). Antibodies to PDH (C54G1, 3205), MCU (D2Z3B, 14997S), Vps34 (D9A5, 4263) were from Cell Signaling Technology (MA, USA). Antibodies to Grp75 (JG1, Ab2799), VDAC1 (20B12AF2, Ab14734), pPDH (EPR12200, Ab177461) and Notch1 (mN1A, 128076) were from Abcam (Cambridge, UK). Antibody to MFN2(XX-1) was from Santa Cruz (Texas, USA). Antibody to IP3R3 was from Merck Millipore (Darmstadt, Germany). Antibodies to Actin (ACTN05, MS-1295-P), Tubulin (MS-581-P0), Normal mouse IgG (NC-1255-P1), and Normal rabbit IgG (NC-100-P1) were from Neomarker (Fremont, CA, USA). Trizol (15596026) and SYBR™ Green Master Mix were from Thermo Scientific (Waltham, MA, USA). PrimeScript 1st strand cDNA Synthesis Kit (6110A) was purchased from Takara Bio (Shiga, Japan).

### Plasmids

Human Bcl-xLRFP plasmid was a gift from Richard J. Youle (National Institutes of Health, Bethesda, MD). NFLGFP was a gift from Freddy Radtke (École Polytechnique Federale de Lausanne (EPFL), Switzerland). pBABE, pBABE-NIC-NLS and pBABE-NIC-NES were kind gifts from B.A. Osborne (University of Massachusetts/Amherst, MA, USA). D1ER and 2mtD3cpv probe were a kind gift from Roger Y Tsien (42, 54).

NIC1-GFP was prepared by sub-cloning NIC1 gifted by J. Aster (Harvard Medical School, Boston) into pEGFP-C1 (BD Clontech, CA, USA). NIC1-RFP has been described earlier (5). sJagged was obtained from Upstate Biotechnology (MA, USA). Human Grp75 Tagged ORF Clone was obtained from Origene (RG201397, MD, USA) and sub-cloned into pBABE vector. Following primers were used for sub-cloning:

NIC1-GFP EcoR1 Forward: 5’-ACTGAATTCTATGCGGCGGCAGCAT-3’
NIC1- GFP BamHI Reverse: 5’-AATGGATCCCTTGAAGGCCTCCGG-3’
Grp75 BamHI Forward: 5’-ATAGGATCCATGATAAGTGCCAGCCGA-3’
Grp75 Sal I Reverse: 5’-ATAGTCGACTTACTGTTTTTCCTCCTTTTGA-3’

Construct sequences were verified by automated Sanger sequencing conducted in-house.

### Isolation of T-Cell Subsets

CD4^+^CD25^+^ Tregs and CD4^+^ naïve T-cells were isolated from murine spleens as previously described (5). For the isolation of ~2 million Tregs, single-cell suspensions from two spleens were generated by dissociating the organs to release cells in PBS, applying gentle but consistent pressure using the back of a syringe plunger. Cells were centrifuged and the red blood cells were lysed by gentle resuspension and vortexing the loosened cell pellet in 1 ml ACK lysis buffer (150 mM NH_4_Cl, 10 mM KHCO_3_, and 0.1 mM Na_2_EDTA) for 1 min. The ACK cell suspension was diluted with excess medium and centrifuged at (500g). The cell pellets was washed with excess medium twice to obtain RBC-free lymphocytes. CD4^+^CD25^+^ Tregs and CD4^+^ naïve T-cells were isolated from the total lymphocyte pool using the Dynabeads FlowComp Mouse CD4^+^CD25^+^ Treg cells Kit (11463D, Invitrogen) and MagniSort Mouse CD4 naïve T-cell enrichment Kit (8804-6824-74, Invitrogen), respectively, following manufacturer’s instructions. Tregs (CD4^+^CD25^+^) and CD4^+^naïve T-cells were activated with 20 μl of magnetic beads coated with antibodies to CD3 and CD28 for 40 h and used in experiments. Isolated CD4^+^CD25^+^ cells were routinely immuno-stained with antibody against Foxp3 and analyzed using Olympus IX70 wide-field fluorescence or Olympus FV3000 confocal microscope. On an average 85-90% or more CD4^+^CD25^+^ cells were Foxp3^+^ when tested by immune-staining.

### Transfections

0.25x10^6^ HEK cells were seeded in tissue culture grade 35 mm dishes (Greiner Bio-one, Kremsmünster, Austria). Transfection with siRNA or plasmids was performed when cultures were 50-60% confluent (24 h post-plating). 100 nM siRNA or plasmids were transfected using Dharmafect and Lipofectamine-2000 or Fugene HD as per the manufacturer’s instructions when cultures were 50–60% confluent (24 h post-plating). Cells transfected with siRNA were incubated for 24–26 h and then harvested by trypsinization and re-plated for transfection with plasmids. Plasmids were transfected using Lipofectamine 2000 or Fugene HD at the following concentrations: NIC1-GFP (2 µg), NIC1-RFP (2 µg), pEGFP-N3 (1 µg), Bcl-xL RFP (1.5 µg), NIC1-NES GFP (2 µg), NIC1-NLS GFP (2 µg) NFL-GFP (2 µg), s-Jagged (2 µg), or 2mtD3cpv (1 µg), pCL-Eco (1.5 µg), pBABE-Grp75 (1.5 µg), pBABE-NIC1-NES (1.5 µg). Total DNA transfected in the different transfection groups was equalized with pcDNA3.

### Retrovirus Transduction

Retrovirus transduction was performed as described (5). Briefly, retroviruses containing the plasmid of interest were packaged in HEK cells using the packaging vector pCL-Eco. HEK cells were co-transfected with pCL-Eco and the plasmid containing gene of interest using X-tremeGENE HP. Viral supernatants were harvested two days post-transfection and concentrated by centrifugation at 21000 g for 1.5 h at 4°C. Viral supernatants were stored at -80°C when not used immediately for a maximum of 14 days. Tregs were activated as described earlier for 24 h with anti-CD3 and CD28 bound magnetic beads in 24 well plates. For retroviral infection, 700 µl of culture medium from Tregs was replaced with an equal volume of concentrated virus in RPMI-CM containing 10 mM HEPES and 8 µg/ml sequabrene, and the plate was centrifuged at 600 g for 90 min at 25°C. Post centrifugation, 700 µl of medium was replaced with RPMI-CM supplemented with 1 µg/ml of IL-2 and cells continued in culture. After a further 24 h, beads were removed by magnetic separation and cells were cultured in RPMI-CM supplemented with IL-2 (1 µg/ml) for another 18–24 h. Cells were harvested and continued (0.5X10^6^/ml) in RPMI-CMcontaining IL-2 (1 µg/ml) and the antibiotic puromycin (1 µg/ml) for 48 h to enrich transfected cells. After 48 h, live cells were collected by centrifugation on histopaque (1.083 g/ml density) at 300 g for 20 min at 25°C and washed twice in RPMI-CM. Cells were cultured for another 24 h in RPMI-CM containing IL-2 (1 μg/ml) and IL-7 (2 ng/ml) and used in functional assays or for metabolic analysis.

### Induction of Apoptosis and Assays for Cellular Damage

Activated Tregs were washed three times with PBS and 0.3x10^6^ cells/well were cultured in a 48 well plate for 24 h. Notch1^-/-^ Tregs infected with pBABE, pBABE NIC-NES and pBABE Grp75 were analyzed as one unit, with data reported in separate figures as indicated in the legends to figures. Cells were harvested and stained with Hoechst 33342 (1 μg/ml), and samples were scored for nuclear damage using fluorescent microscope (Olympus BX-60). Samples were blinded for the experimenter and approximately 200 cells in 5 random fields were scored for apoptotic damage. 0.5x10^6^ activated Tregs were incubated with DiOC_6_ (40 nM) diluted in PBS for 10 min at 37°C protected from light. After 10 min, cells were given three washes with pre-warmed (37°C) PBS to remove excess dye and immediately analyzed using BD FACS Fortessa flow cytometer.

### RT PCR Analysis

3x10^6^ Tregs cells were lysed in 1ml of TRIzol and RNA isolation was performed according to the manufacturer’s instructions. 1 µg RNA was used for cDNA synthesis using PrimeScript 1^st^ strand cDNA Synthesis Kit (Takara Bio). cDNA was diluted in 1:5 ratio and real-time PCR was performed using Maxima™ SYBR Green qPCR Master Mix and Bio-Rad CFX96 Touch™ Real-Time PCR Detection System. Relative change in transcript levels was calculated using 2^–ΔΔCt^ method using glyceraldehyde 3-phosphate dehydrogenase (GAPDH) or Actin as reference gene.

Primers used for RT PCR against Human genes: Forward (5’–3’); Reverse (5’–3’)
GAPDH: TGCACCACCAACTGCTTAGC; GGCATGGACTGTGGTCATGAG
MCU: ACCGGACGGTACACCAGAG; GATAGGCTTGAGTGTGAACTGAC
VDAC1: CTCCCACATACGCCGATCTT; GCCGTAGCCCTTGGTGAAG
Grp75: TGTGGCCTTTACAGCAGATG; ATCACCCGAAGCACATTCAG
IP3R3: CTGGTGTTCTTTGTCAGCGA; TTCTGCTCCCTCATCAGCTT
Primers used for RT PCR against Murine gene: Forward (5’–3’); Reverse (5’–3’)
Notch1: GGAAGCACCCTTTAGGTTGGA; AGTGGTCCAGGGTGTGAGTGT
Actin: TGGGTCAGAAGGACTCCTATG; CAGGCAGCTCATAGCTCTTCT
MCU: GAGCCGCATATTGCAGTACGGT; AAACACGCCGACTGAGTCAGAG
VDAC1: AGTGACCCAGAGCAACTTCGCA; CAGGCGAGATTGACAGCAGTCT
Grp75: GTTGGTATGCCAGCAAAACGGC; CAAGCATCACCATTGGAGGCAC
IP3R3: GCAACCACATCTGGACGCTCTT; AGAAGGCACTGATGGTGTCCAG

### Passive Store Depletion (PSD) Assay

Measures of Ca^2+^ dynamics were all performed in Ca^2+^-free medium. HEK cells, pre-treated with the siRNA, were transfected with NIC1-RFP or control RFP using Fugene HD as described above. Cells expressing RFP or NIC1-RFP were loaded with 3.5 μM of Fluo4-AM or Indo-1-AM dye, 0.002% Pluronic F-127 in DMEM-CM for 20 min in humidified 5% CO_2_, 37 °C incubator in the dark. After incubation, cells were washed with PBS and imaged in calcium free media. Time lapse images of cells loaded with Fluo4-AM were acquired every 10 s for 6 min using 488 nm excitation and 505 nm emission on an Olympus IX70 wide field fluorescence microscope with 60X, NA 1.4 oil objective before and after addition of 2 µM TG. Time lapse images of cells loaded with Indo-1 dye were acquired every 10 s for 6 min using Epifluor optics (Nikon TE 2000 inverted wide field microscope) with 60X, NA 1.4 oil objective. Indo-1 was excited using 365/10 exciter and emission collected by D405/30 and D485/25. The images were analyzed and quantified for the intensities using Image J software.

### Assessment of Free Calcium in ER and Mitochondria

Analysis of free calcium in ER and mitochondria were performed using FRET based D1ER and 2mtD3cpv probes, respectively. 0.2x10^6^ HEK cells were plated onto the cut dishes with microscopy grade coverslip. Next day, cells were transfected with 2mtD3cpv or D1ER with NIC1-RFP, Bcl-xL-RFP, or RFP using Fugene-6 HD. 36 h post transfection, the cells were imaged using Zeiss LSM 510 Meta, Plan apochromat 63X oil, NA 1.42 with FRET module. Probes were excited with 458 nm laser and the emission was collected by BP (470-500) for CFP and BP (510-550) for FRET. The images were captured in time-lapse mode for 3-4 min. YFP and CFP fluorescence were quantified after removing the background signal by the thresholding the image using Image J software.

### Seahorse-Based Analysis

Oxygen consumption rate (OCR) and extracellular acidification rate (ECAR) were measured using Seahorse XFe24 bioanalyzer. Seahorse culture plate were coated with 1 mg/ml poly-D lysine in PBS for 30 min at room temperature and then washed twice with PBS. ~0.3x10^6^ activated Tregs or T-effectors were plated onto the poly-D lysine coated Seahorse culture plate in Seahorse RPMI XF media containing 2 mM L-glutamine, 10 mM sodium pyruvate and 10 mM glucose for OCR analysis or Seahorse RPMI XF media containing 2 mM L-glutamine for ECAR analysis. Cells were incubated for 45 min at 37°C in a humidified non-CO_2_ incubator before OCR and ECAR analysis. OCR and ECAR were measured at baseline and in response to the following inhibitors: 1.25 µM oligomycin, 1 µM FCCP, and 1 µM rotenone and antimycin A for OCR analysis and 10 mM glucose, 1.25 µM oligomycin, 50 mM 2-deoxyglucose for ECAR. Four wells in each plate containing Seahorse RPMI XF media were used as background. Notch1^-/-^ Tregs infected with pBABE, pBABE NIC-NES and pBABE Grp75 were analyzed together, with data reported in separate figures as indicated in figure legends. Basal respiration = OCR before oligomycin addition– Last OCR after Rotenone + AntimycinA addition. Maximum respiration= Maximum OCR after FCCP addition – Last OCR after Rotenone + AntimycinA addition.

### Immunofluorescence Analysis

Cells were plated on poly-D-lysine coated dishes (1 mg/ml poly-D-lysine in PBS coated for 15 min at room temperature) for 10 min and fixed with 2% paraformaldehyde (freshly reconstituted) for 20 min in the dark at room temperature. For Grp75 and Foxp3, cells were permeabilized using 0.2% Triton-X 100 and 0.2% NP-40, respectively, for 10 min at room temperature. Cells were incubated with Grp75 (1:100) antibody diluted in 5% BSA in PBS or with 5% BSA in PBS for secondary control and incubated overnight at 4°C. The next day, cells were washed two times with PBS and incubated with secondary fluorescence-conjugated antibody (1:500) for 1 h in the dark at room temperature. Cells were washed twice with PBS and stained with Hoechst 33342 (1 μg/ml) for 10 min and ten random fields were imaged using Olympus FV3000 as Z-stacks (1.0 µm, 3X zoom); Plan-Apochromat 63X NA 1.35 oil-immersion objective. Images were processed to remove background based on secondary controls.

### Immunoprecipitation and Western Blotting

Activated 4x10^6^ Tregs cells cultured for 6 h without IL-2 in RPMI-CM, were lysed for 30 min at 4°C on a rotational cell mixer in RIPA buffer containing 1% NP-40, 5% glycerol, 50 mM Tris, 1 mM NaCl, and 1 mM EDTA and supplemented with aprotinin, leupeptin, and pepstatin (2 μg/ml each), 10 μM MG132, 1 mM PMSF, 1 mM NaF, and 1 mM Na_3_VO_4_. After lysis, lysates were centrifuged at 1500 g for 5 min to remove cell debris, and supernatants were incubated with antibody (6 μg) or IgG control (6 μg) for 16-18 h at 4°C on a rotational cell mixer. The Immune complexes were then incubated with Sepharose G/A plus bead slurry for 3 h at 4°C on a rotational cell mixer. Beads bound to complexes were washed three times by adding ice-cold PBS followed by centrifugation at 300 g for 2 min. Finally, beads were boiled in SDS lysis buffer containing protease inhibitors for 10 min and analyzed by western blot. 0.3 x 10^6^ Tregs or 0.1x10^6^ HEK cells were pelleted down by centrifugation at 1000 g for 5 min at room temperature and 25 µl of SDS lysis buffer (2% SDS, 10% glycerol, 0.002% bromophenol blue, 200 mM DTT and 50 mM Tris-Cl pH 6.8) containing a protease inhibitor cocktail - aprotinin, leupeptin and pepstatin (2 μg/ml each), 1 mM PMSF, 1 mM NaF, 1 mM Na_3_VO_4_ and 10 μM MG132 was added to the pellet. The tube was vortexed for 20-30 s and incubated at 100°C for 10 min. Cell lysates were immediately resolved by SDS-PAGE and transferred to nitrocellulose membrane (GE Healthcare, Chicago, USA) and blocked with 5% non-fat dried milk in Tris-buffered saline–Tween 20 (TBST), and incubated overnight at 4°C with primary antibodies at the following concentration: Grp75 (1:500), Notch1 (1:500), VDAC1 (1:500), Vps34 (1:500), MCU (1:500), pPDH (1:500), PDH (1:500), Actin (1:1000) and Tubulin (1:1000) diluted in 5% non fat dried milk in TBST. After incubation with primary antibodies, membranes were washed three times with TBST followed by incubation with horseradish peroxidase–conjugated secondary antibody (1:1000 dilutions) for 1 h at room temperature. After incubation with secondary antibody, membranes were washed three times with TBST. Membranes were developed using Super Signal West Dura substrate (Thermo Scientific), and images were acquired using iBright FL1000 Invitrogen. Densitometric analysis of western blots was performed using Image J software.

### Statistical Analysis

Data are represented as mean ± standard deviation (Mean ± SD) derived for two or three independent experiments. Statistical significance was measured using unpaired student’s t-test and p-values ≤0.05, ≤0.01 and ≤0.001 were considered to be statistically significant. p-value >0.05 were considered non-significant (ns).

### Ethical Approval

All experimental protocols involving mice were approved by the Institutional Animal Ethics Committee (INS-IAE-2019/07(R1)) and complied with the norms of the Committee for the Purpose of Control and Supervision of Experiments on Animals, Government of India.

## Results

### Notch1 Signaling Modulates Mitochondria Metabolism in Tregs

**Figure 1:**
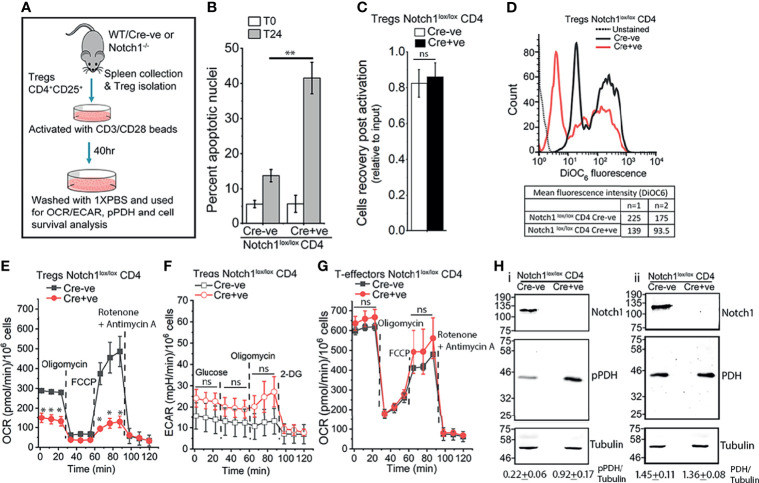
Notch1 signaling regulates mitochondrial metabolism in Tregs. **(A)** Experimental protocol for Tregs activation. **(B)** Percent apoptotic nuclei in activated Notch1^+/+^ (Cre-ve; *Notch1* ^lox/lox^) and Notch1^-/-^ (Cre+ve; *Cd4-Cre::Notch1* ^lox/lox^) Tregs at onset (T0), and after 24 h culture (T24) in medium without IL-2. **(C)** Cell recoveries in the two genotypes at the end of the 40 h activation protocol. **(D)** Representative histogram overlay and mean fluorescent intensity (MFI) of DioC_6_ in freshly activated Notch1^+/+^ (black) and Notch1^-/-^ (red) Tregs. The dotted line indicates unstained Tregs. **(E)** Oxygen consumption rate (OCR) in activated Notch1^+/+^ (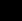) and Notch1^-/-^ (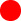) Tregs at baseline and following sequential addition of 1.25 μM Oligomycin, 1 μM FCCP and 1 μM Rotenone + 1 μM Antimycin A. **(F)** Extracellular acidification rate (ECAR) in activated Notch1^+/+^ (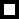) and Notch1^-/-^ (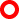) Tregs at baseline and in response to sequential treatment with 10 mM glucose, 1.25 μM oligomycin and 50 mM 2-deoxyglucose. **(G)** OCR measured in CD4^+^ naïve T-cells isolated from Notch1^+/+^ (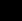) or Notch1^-/-^ (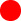) mice and activated as shown in A, in response to 1.25 μM Oligomycin, 1 μM FCCP and 1 μM Rotenone + 1 μM Antimycin A. **(H)** Representative immunoblots of whole cell lysates from activated Notch1^+/+^ and Notch1^-/-^ Tregs. Lysates were divided into two and analyzed in parallel. Membranes were probed either for pPDH, Notch1, and Tubulin **(H–I)** or PDH, Notch1, and Tubulin **(H-ii)**. The immunoblots are representative of three independent experiments. The densitometry analysis (Mean ± SD from three independent experiments) of pPDH and PDH relative to Tubulin are shown below the immunoblots. The same pool of activated Tregs was tested in **(E, F)**. In **(E–G)**, plots shown as mean ± SD of readings in triplicate wells and are representative of two separate experiments. * and ** denote significant differences with p-value ≤ 0.05 and ≤0.01 respectively, ns: not significant, examined using the unpaired student’s t-test.

Tregs constitutively express the cytokine Interleukin-2 (IL-2) receptor, CD25, and the transcription factor Foxp3 (55, 56). We have shown that ligand-dependent Notch1 activity in activated Tregs, regulates their IL-2-independent survival (5). Thus, unlike cells isolated from *Notch1^lox/lox^* Cre-ve, mice, CD4^+^Foxp3^+^CD25^+^ cells isolated from mice with a targeted ablation of *Notch1* (*Cd4-Cre::Notch1* ^lox/lox^ mice), in the mature T-cell compartment, will undergo apoptosis if cultured without cytokine (**Figures 1A, B**). However, the recovery of viable (Cre-ve) Notch1^+/+^ and (Cre+ve) Notch1^-/-^ Tregs from activation protocol is comparable (**Figure 1C**), ruling out deficits in responses to T-cell receptor dependent activation. Nonetheless, small but consistent differences in mitochondrial transmembrane potential between activated Tregs of the two genotypes (**Figure 1D**), prompted an assessment of metabolic profiles of Tregs. Seahorse based analysis of (Cre-ve) Notch1^+/+^ and (Cre+ve) Notch1^-/-^ Tregs revealed that as compared to (Cre-ve) Notch1^+/+^ cells, oxidative phosphorylation (Oxphos), was blunted in (Cre+ve) Notch1^-/-^ Tregs (**Figure 1E**, red trace at baseline). Notch1^-/-^ Tregs also had reduced respiratory reserve, indicated by the poor recovery following FCCP treatment (**Figure 1E**). Notably, glycolysis was comparable, with the Notch1^-/-^ cells, presenting marginally elevated activity relative to Notch1^+/+^ cells (**Figure 1F**). On the other hand, when T-effector subsets, generated by activating CD4^+^ naïve T-cells in culture (**Figure 1A**), from the two genotypes, were compared, metabolic profiles of (Cre-ve) Notch1^+/+^ and (Cre+ve) Notch1^-/-^ T-effectors were comparable (**Figure 1G** and **Supplementary Figure S1**). Thus, Notch1 regulation of mitochondrial metabolism is not a generalized feature of the T-cell lineage.

Calcium regulation of mitochondrial metabolic activity is well established (40). Hence, we compared calcium-sensitive phosphorylation of the enzyme Pyruvate Dehydrogenase (PDH) in the two genotypes. Substantially higher levels of phosphorylated (p)PDH, were detected by western blot analysis of cell lysates in (Cre+ve) Notch1^-/-^ Tregs (**Figure 1Hi**, lane 2) as compared to (Cre-ve) Notch1^+/+^ (**Figure 1Hi**, lane 1), which suggests reduced level of free calcium in mitochondria. Total PDH is comparable in the cells of the two genotypes (**Figure 1Hii**, row 2). In order to directly assess organelle calcium and explore underlying molecular interactions, we used the mammalian cell line HEK, in which Notch1 activated signaling has been previously characterized (6–8).

### NIC1 Signaling Regulates ER Calcium`

**Figure 2:**
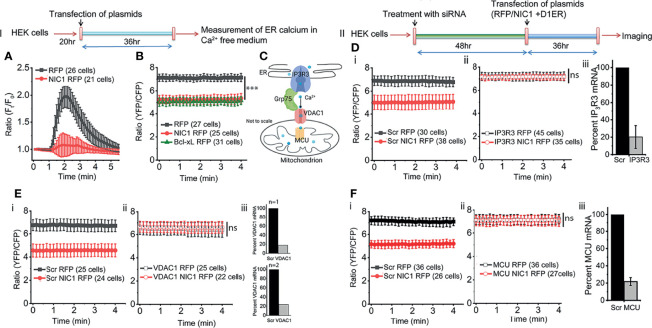
NIC1 signaling regulates ER calcium levels. Schematic **(I)** Timeline of experiments assessing changes in ER calcium levels. Schematic **(II)** Timeline of experiments, in cells treated with siRNA before assessing changes in ER calcium levels. **(A)** Fluo4 fluorescence in HEK cells expressing NIC1-RFP (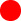) or RFP (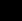), at time-t (F_t_) relative to onset (F_0_) measured in calcium free medium at baseline and in response to 2 μM TG. **(B)** YFP/CFP ratio in HEK cells co-transfected with D1ER and NIC1-RFP (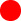) or, Bcl-xL-RFP (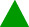) or, RFP (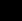) and cultured for 36 h before imaging. **(C)** Schematic (not to scale) depicting - IP3R3, Grp75, VDAC1 and MCU- in the context of ER and mitochondria. **(D–F)** Ratio of YFP/CFP fluorescence in HEK cells, imaged 36 h after co-transfection with D1ER and NIC1-RFP (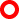) or RFP (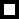), in cells pre-treated with siRNA to IP3R3 **(D-ii)** or VDAC1 **(E-ii)** or MCU **(F-ii)** or scrambled control (**D-F-i**, NIC1-RFP (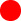) and RFP (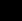). **(D–F iii)** Percent mRNA levels of the genes as shown in panels, in cells treated with siRNA to IP3R3 (**D**, N=3) or VDAC1 (**E**, N=2) or MCU (**F**, N=3) and scrambled control. Data plotted as mean ± SD of the indicated number of cells in 6-12 fields across three **(A, D, F)** or two **(B, D)** independent experiments. *** indicates significant difference at all time points with p-values ≤0.001 and ns: not significant, examined using unpaired student’s t-test.

The ER lumen is the major store of free calcium (57). The passive store depletion (PSD) assay (58) was deployed to assess the effects of Notch1 activity, if any, on the release of calcium into the cytoplasm in response to an ER destabilizing trigger. HEK cells expressing processed NIC1 or a control vector were loaded with the calcium sensitive dye Fluo-4, and assessed in the PSD assay, performed in calcium free media (**Figure 2**, Schematic-I). The addition of Thapsigargin (TG), the ER calcium re-uptake inhibitor, resulted in a rapid, characteristic rise of calcium (increase in fluorescence) in the cytoplasm of control (RFP expressing) cells (**Figure 2A**, black trace). The dissipation that follows, is a result of the release of calcium into the extracellular medium (59, 60). However, only a marginal increase in fluorescence - release of calcium from the ER store - was observed in cells expressing NIC1, following the addition of TG (**Figure 2A**, red trace). This can result from reduced calcium in the ER lumen or a deficit in the machinery by which calcium is released into the cytoplasm from the ER.

Next, an assay that provides direct readout of levels of free calcium in the ER lumen was performed. This assay compared cells expressing the ER localized FRET-based calcium sensor, D1ER (42) co-transfected with NIC1-RFP or with RFP (**Figure 2**, Schematic-II). At steady state, i.e. in cells held in culture with no additional perturbation, D1ER fluorescence, indicative of free calcium levels, was lower (***p<0.001, student’s t-test) in cells expressing NIC1-RFP than in cells expressing RFP (**Figure 2B**, compare black vs red trace). We recapitulated published data (54, 61) of reduced ER calcium levels (D1ER-fluorescence), in cells expressing the anti-apoptotic protein Bcl-xL (**Figure 2B**, green trace) relative to the control group. This observation was consistent with the results of the PSD analysis and argued against a major defect in calcium release from the ER.

In the experiments that follow the involvement of molecular complexes that regulate inter-organelle movement of calcium were examined for the modulation, if any, on Notch1. The transfer of calcium between ER and mitochondria has been demonstrated in many cell types and molecules such as IP3R3 (calcium release from the ER), Grp75 and VDAC1, and MCU (mitochondrial uptake of calcium), are implicated in this transfer (**Figure 2C**). Changes if any, in levels of ER calcium were compared in cells transfected with NIC1 or a control vector (**Figure 2Di**), against the cellular background of RNAi mediated ablation of IP3R3 or VDAC1 or MCU, or the scrambled control. In summary, ablation of any one of these proteins, restored levels of ER calcium in NIC1 expressing cells, to levels of the control group (**Figures 2Dii-Fii**). Notably, there was no change in ER calcium in RFP expressing cells (control) treated with different siRNA (**Figures 2D–F**). The requirement of these molecules was also confirmed using the PSD assay in cells expressing NIC1 or the control vector (**Supplementary Figure S2A–C**). Collectively, these experiments indicated that NIC1 modulates intracellular calcium dynamics and this is dependent on molecules controlling calcium release from the ER store (IP3R3) and uptake by mitochondria (VDAC1 and MCU). Next, the status of free calcium in mitochondria was assessed.

### NIC1 Signaling Maintains Mitochondrial Calcium Levels

**Figure 3:**
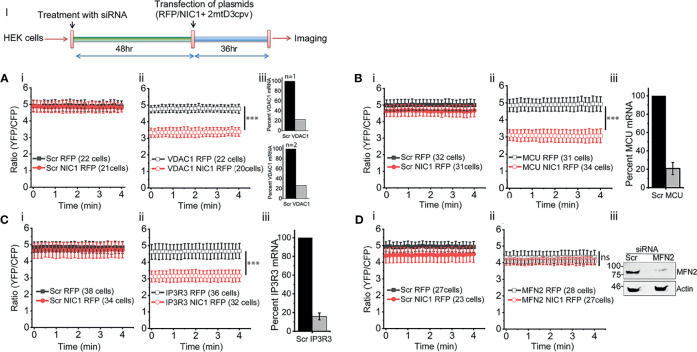
NIC1 signaling maintains mitochondrial calcium levels. Schematic **(I)** Timeline of experiments, for the assessment of mitochondrial calcium levels. **(A–D)** Ratio of YFP/CFP fluorescence in HEK cells, imaged 36 h after co-transfection with 2mtD3cpv and NIC1-RFP (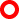) or RFP (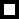) in cells pre-treated with siRNA to VDAC1 **(A-ii)** or MCU **(B-ii)**, IP3R3 **(C-ii)** or MFN2 **(D-ii)** or scrambled control (**A-D-i**, NIC1-RFP (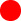) and RFP (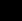). **(A-C iii)** Percent mRNA of the genes as shown in panels, in cells treated with siRNA to VDAC1 (**A**, N=2) or MCU (**B**, N=3) or IP3R3 (**C**, N=3) and scrambled control. **(D-iii)** Representative immunoblot of whole cell lysates from cells treated with siRNA to MFN2 or scrambled control probed for MFN2 and Actin. Data plotted as mean ± SD of the indicated number of cells in 6-12 fields across two **(A, D)** or three **(B, C)** experiments. *** indicates significant difference at any time point with p ≤ 0.001 and ns: not significant, examined using the unpaired student’s t-test.

To assess calcium levels in mitochondria the FRET-based calcium sensor, 2mtD3cpv, which localizes to mitochondria under the control of an addressing tag, was used (54). The sensor was co-transfected in cells with NIC1 or RFP (control vector) and FRET measurements were performed (**Figure 3**, Schematic I). The unexpected observation in these experiments was that unlike the observations in the ER, mitochondrial calcium levels were comparable in control (RFP) or NIC1-RFP expressing cells (**Figures 3Ai–Di**). A control assay to show that the probe can detect changes in calcium levels was also performed and confirmed its function (**Supplementary Figure S3**). However, the probe was not sensitive to small changes in calcium levels, such as those triggered by the addition of TG to cells (data not shown). Nonetheless, changes in mitochondrial free calcium were readily apparent in NIC1 expressing cells, following the RNAi-mediated ablation of VDAC1, or MCU or IP3R3. The ablation of any one of these proteins, resulted in a drop in the levels of mitochondrial free calcium (**Figures 3Aii–Cii**) in cells co-expressing NIC1, indicating that Notch1 can modulate mitochondrial calcium dynamics. We next tested the effect of ablating Mitofusin (MFN)2, which regulates mitochondrial homeostasis (62), and modulates NIC1-mediated anti-apoptotic activity (7). Following the ablation of MFN2 mitochondrial calcium levels remained comparable in cells expressing NIC1or the control vector (**Figure 3D**). Together, these results are consistent with a role for Notch1 in modulating mitochondrial calcium uptake, *via* molecular complexes that bridge ER and mitochondria. Thus, calcium released from the ER is taken up by mitochondria (24, 38, 63–65), with uptake of calcium inhibited if proteins such as VDAC1 and MCU are depleted. Therefore, blocking mitochondrial uptake of calcium from the cytoplasm would lower mitochondrial calcium levels, as observed in the NIC1 expressing cells in the experiments.

### Ligand-Dependent, Non-Nuclear NIC1 Signaling Regulates Calcium Levels

**Figure 4:**
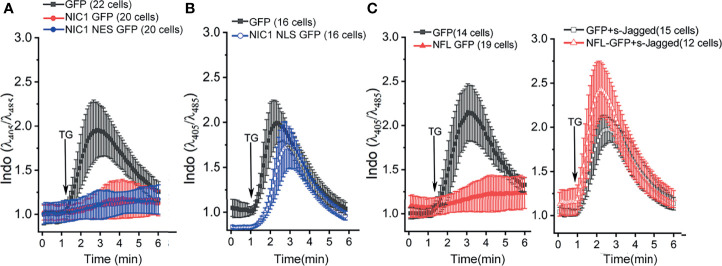
Ligand-dependent, non-nuclear NIC1 signaling regulates ER calcium levels. **(A, B)** Ratio of intracellular Indo-1 dye fluorescence at 405 nm and 485 nm at baseline and in response to 2 µM TG in HEK cells measured in calcium-free medium, and imaged 36 h post-transfection with GFP (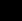), NIC1 GFP (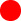) or NIC1-NES GFP (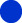) **(A)** or NIC1-NLS GFP (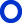) **(B)**. **(C)** Ratio of intracellular Indo-1 dye fluorescence at 405 nm and 485 nm wavelength at baseline and in response to 2 µM TG addition in HEK cells measured in calcium-free medium, and imaged 36 h post-transfection with plasmids encoding for GFP (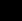), NFL GFP (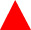), GFP and sJagged (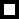) or NFL GFP and sJagged (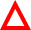). Data plotted as mean ± SD of the total number of cells indicated in parentheses, from three **(A)** and two **(B, C)** experiments.

Notch1-mediated anti-apoptotic activity, albeit executed from the cytoplasm, requires ligand-mediated processing (5–8). To assess if this was also true for the regulation of calcium dynamics, we expressed NIC1, tagged to addressing constructs that spatially restrict the protein to the cytoplasm (NIC1 tagged to **N**uclear **E**xport **S**equence, NIC1-NES) or restricting it to the nucleus (NIC1 tagged to **N**uclear **L**ocalization **S**equence, NIC1-NLS). NIC1_NES or NIC1_NLS were expressed in HEK cells and assessed in the PSD assay. Cells expressing NIC1-NES (**Figure 4A**, blue trace) overlapped the patterns of cells expressing NIC1 (**Figure 4A**, red trace), with little or no signals of cytoplasmic calcium following exposure to TG. On the other hand, in cells expressing NIC1-NLS, the release of calcium was comparable to cells that expressed GFP alone (**Figure 4B**, blue trace), indicating that enforced nuclear localization restricted this aspect of NIC1 activity. NIC1-NLS activity is confirmed in assays of transcription (66). To test ligand-dependence of Notch1 activity the following was done. HEK cells expressing the full-length form of the Notch1 receptor (NFL) and those co-expressed with a soluble form of extracellular domain of Jagged (sJagged), which functions as a dominant negative and blocks the processing and cleavage of Notch1 were tested (6). In cells expressing NFL, fluorescence is blunted in response to TG (**Figure 4C**, red trace) as seen with cells expressing NIC1 (**Figure 4A**). However, in cells expressing tagged and NFL, the profile of calcium release and its dissipation were comparable to cells expressing GFP (**Figure 4C**), indicating that Notch1 processing to NIC1 was required for this function. Together, the experiments show that NIC1 activity from the cytoplasm modulates calcium homeostasis.

### NIC1-NES-Mediated Treg Survival Is Dependent on Calcium Signaling

**Figure 5:**
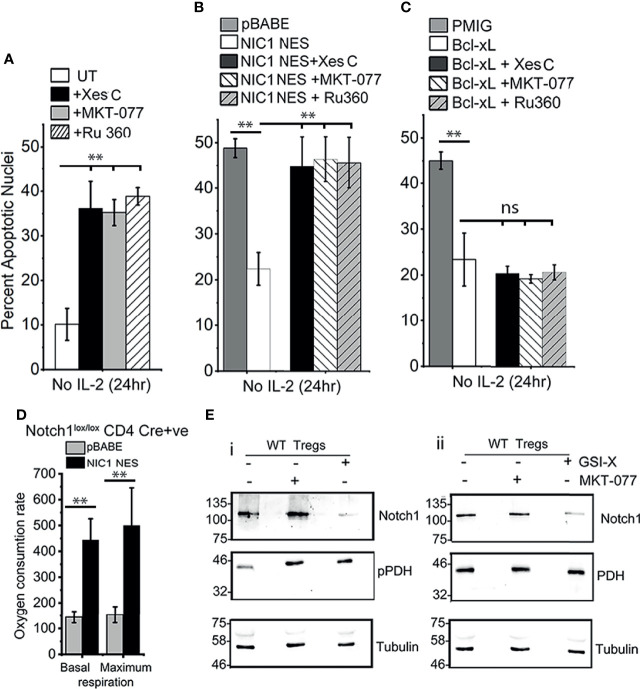
NIC1-mediated Treg survival requires IP3Rs, Grp75 and MCU. **(A)** Percent apoptotic nuclei in activated WT Tregs cultured without IL-2 for 24 h with vehicle control (UT) or, 5 μM Xestospongin C or, 10 μM MKT-077 or, 10 μM Ru360. **(B, C)** Percent apoptotic nuclei in Notch1^-/-^ Tregs transduced with pBABE or NIC1-NES **(B)** or pMIG or Bcl-xL**(C),** and cultured without IL-2 for 24 h with vehicle control, or 5 μM Xestospongin C or, 10 μM MKT-077 or, 10 μM Ru360. **(D)** Basal and maximum OCR, computed as described in methods, in Notch1^-/-^ Tregs transduced with pBABE or NIC1-NES. **(E)** Immunoblot of whole cell lysates prepared from activated WT Tregs cells cultured with or without 10 μM GSI-X or 10 μM MKT-077 for 8 h and the samples run in duplicate. Membrane was probed either for (p)PDH, Notch1 and Tubulin **(E-i)** or PDH, Notch1 and Tubulin **(E-ii)**. The immunoblot is representative of two independent experiments. Data are mean ± SD of three independent experiments **(A–C)** and readings in 4 wells from two independent experiments **(D).** ** indicates significant difference with p-value ≤0.01 and ns, not significant, examined using unpaired student’s t-test.

With these leads from analysis in the HEK cell line, we next assessed Notch1-mediated Treg survival for dependency on molecules regulating inter-organelle calcium transfer. As shown in **Figure 5A**, Notch1-dependent wildtype (WT) Tregs survival (in cells cultured without IL-2), was compromised by the inhibition of IP3R3 (Xestospongin C), or, Grp75 (MKT-077) or MCU (Ru-360). The inhibitors were without effect on Tregs viability, when cultured in the presence of IL-2 (**Supplementary Figure S4**). The inhibitors were also tested in Cre+ve Notch1^-/-^ Tregs transduced with NIC1-NES (**Figure 5B**) or Bcl-xL (**Figure 5C**), since both confer IL-2-independent survival in these cells. The protective effect of NIC1-NES in Notch1^-/-^ Tregs was attenuated by the inhibitors, and was comparable to cells transduced with the control vector pBABE (**Figure 5B**). In cells expressing Bcl-xL, the inhibitors were without effect (**Figure 5C**), ruling out generalized toxicity of these chemicals and indicating specific regulation of Notch1 mediated survival.

Seahorse-based analysis of NIC-NES expressing (Cre+ve) Notch1^-/-^ Tregs, showed a restoration of mitochondrial oxphos function (**Figure 5D**), which was comparable to (Cre-ve) Notch1^+/+^ Tregs shown earlier in the study (**Figure 1E**). We next tested the effects of inhibiting NIC1 activity or that of Grp75 on calcium dependent phosphorylation of PDH. To this end, levels of phospho-PDH were compared in wildtype (WT) Tregs cultured as such, or in the presence of γ-Secretase Inhibitor (GSI) to block Notch1 processing or MKT-077. As shown, phosphorylation of PDH is elevated in cells treated with GSI-X or MKT-077 (**Figure 5Ei**, lanes 2 and lane 3), compared to vehicle control treated groups (**Figure 5Ei**, lane 1). Levels of the PDH enzyme remain unchanged (**Figure 5Eii**, row 2). Expectedly, treatment with GSI-X (and not MKT-077), resulted in a loss of processed Notch1 (**Figure 5Ei, ii**).

### Notch1-Grp75 Interactions in Tregs

**Figure 6:**
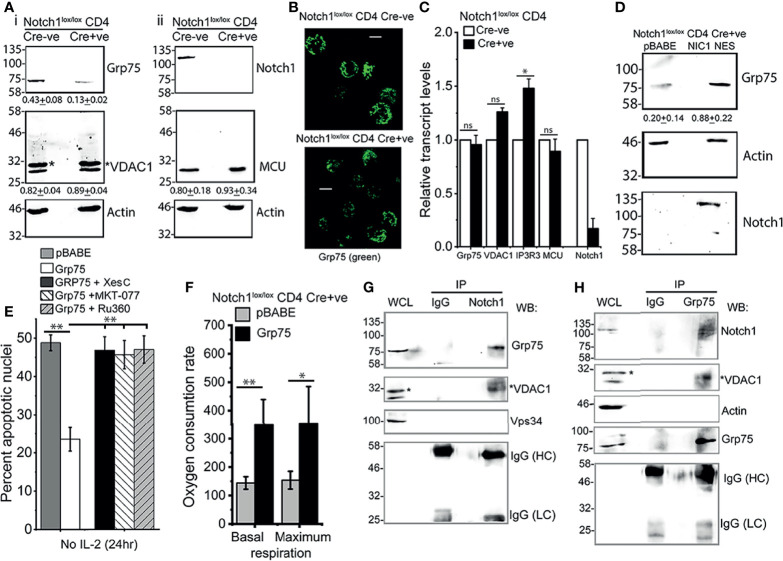
Notch1 activity regulates Grp75 protein levels. **(A)** Immunoblots of whole cell lysates prepared from activated Notch1^+/+^ (Cre-ve) and Notch1^-/-^ (Cre+ve; *Cd4-Cre::Notch1* ^lox/lox^) Tregs, run in duplicate. Membranes were sequentially probed for Grp75, VADC1 (*VDAC1 band), and Actin **(A-i)** or Notch1, MCU and Actin (A-ii). Mean ± SD values below are the densitometry analysis of Grp75, VDAC1 and MCU relative to Actin. **(B)** Representative Z-projected confocal images of Notch1^+/+^ and Notch1^-/-^ Tregs immune-stained with an antibody to Grp75 (green). scale bar: 5 μm. Images are representative of 106 Notch1^+/+^ Tregs and 82 Notch1^-/-^ Tregs. **(C)** Relative transcript levels of indicated genes in Notch1^+/+^ and Notch1^-/-^ activated Tregs. **(D)** Immunoblots of cell lysates from Notch1^-/-^Tregs transduced with pBABE or NIC1-NES probed for Notch1, Grp75 and Actin. Mean ± SD values below are the densitometry analysis of Grp75 relative to Actin. **(E)** Percent apoptotic nuclei in Notch1^-/-^ Tregs transduced with pBABE or Grp75, cultured without IL-2 for 24 h with 5μM Xestospongin C or, 10 μM MKT-077 or, 10 μM Ru360. **(F)** Basal and maximum OCR in Notch1^-/-^ Tregs transduced with pBABE or Grp75. Control (pBABE) condition in panels E and F are common **Figures 5B, D** as these were tested in the same experiment, as described in methods**. (G, H)** Cell lysates of WT activated Tregs cultured for 6 h without IL-2 were subject to immunoprecipitation using an antibody to Notch1 **(G)** or Grp75 **(H)** or, IgG (Isotype control), and associated proteins analyzed by western blotting for Grp75, VDAC1 (*shows VDAC1), Notch1, Vps34, Actin and IgG (Isotype control). Immunoblots are representative of three independent experiments **(A)** or, two independent experiments **(D, G, H)**. Data show the mean ± SD of three independent experiments **(C, E)** and readings in 4 wells from two independent experiments **(F).** * and ** indicates significant difference with p-value ≤0.05 and ≤0.01 respectively, and ns: not significant, examined using unpaired student’s t-test.

In further characterization, immunoblot analysis of Grp75, VDAC1, and MCU proteins in (Cre-ve), Notch1^+/+^ and (Cre+ve) Notch1^-/-^ activated Tregs, revealed that the levels of Grp75 protein were substantially reduced in Notch1^-/-^ Tregs (**Figure 6A**). Immunostaining for Grp75 in fixed cells confirmed the difference (**Figure 6B**). However, there was no change in Grp75 transcript levels in Cre+ve Notch1^-/-^ cells relative to Cre-ve Notch1^+/+^ Tregs (**Figure 6C**). Further, we observed that the levels of Grp75 are restored in (Cre+ve) Notch1^-/-^ cells expressing NIC-NES (**Figure 6D**), to levels close to Tregs expressing the full complement of Notch1, suggesting that the regulation is likely post-transcriptional.

The overexpression of Grp75 by retroviral transfection in (Cre+ve) Notch1^-/-^ cells restored IL-2-independent survival (**Figure 6E**), which was abrogated by chemical inhibition of IP3R3 or Grp75 or MCU (**Figure 6E**). In (Cre+ve) Notch1^-/-^ Tregs, expressing Grp75, basal and maximum levels of mitochondrial respiration, assessed by Seahorse-based analysis, also showed improvement, albeit not to the levels of NIC-NES expressing cells tested in the same assay (**Figure 6F**). These data suggest a role for Grp75 as an intermediate in the Notch1 activated pathway in Tregs.

Hence, we next examined if these molecules were associated in immunecomplexes by immunoprecipitation of endogenous proteins in wildtype Tregs. An antibody to Notch1 but not control IgG, immunoprecipitated Grp75, and the associated protein VDAC1 (**Figure 6G**, lane 3 vs lane 2). These results were confirmed by reverse immunoprecipitation with an antibody to Grp75, and the detection of both Notch1 and VDAC1 in the associated complex (**Figure 6H**, lane 3). The specificity of these interactions was confirmed by the absence of proteins in the control IgG IP (**Figures 6G, H**, lane 2) as well as the exclusion of Vps34 or actin, from the Notch1 immunoprecipitated complex (**Figures 6G, H**). In related experiments, NIC1 did not immuneprecipitate IP3R3 in immunecomplexes that include Grp75 (**Supplementary Figure S5**). IP3R3 is a large molecular weight (~300kDa) protein and it was relatively difficult to detect by immunoblotting in Tregs. Hence, although not detected in immune-complexes in our experiments, this does not definitively rule out the dynamic participation of IP3R3 in immune-complexes formed by NIC1.

## Discussion

**Figure 7:**
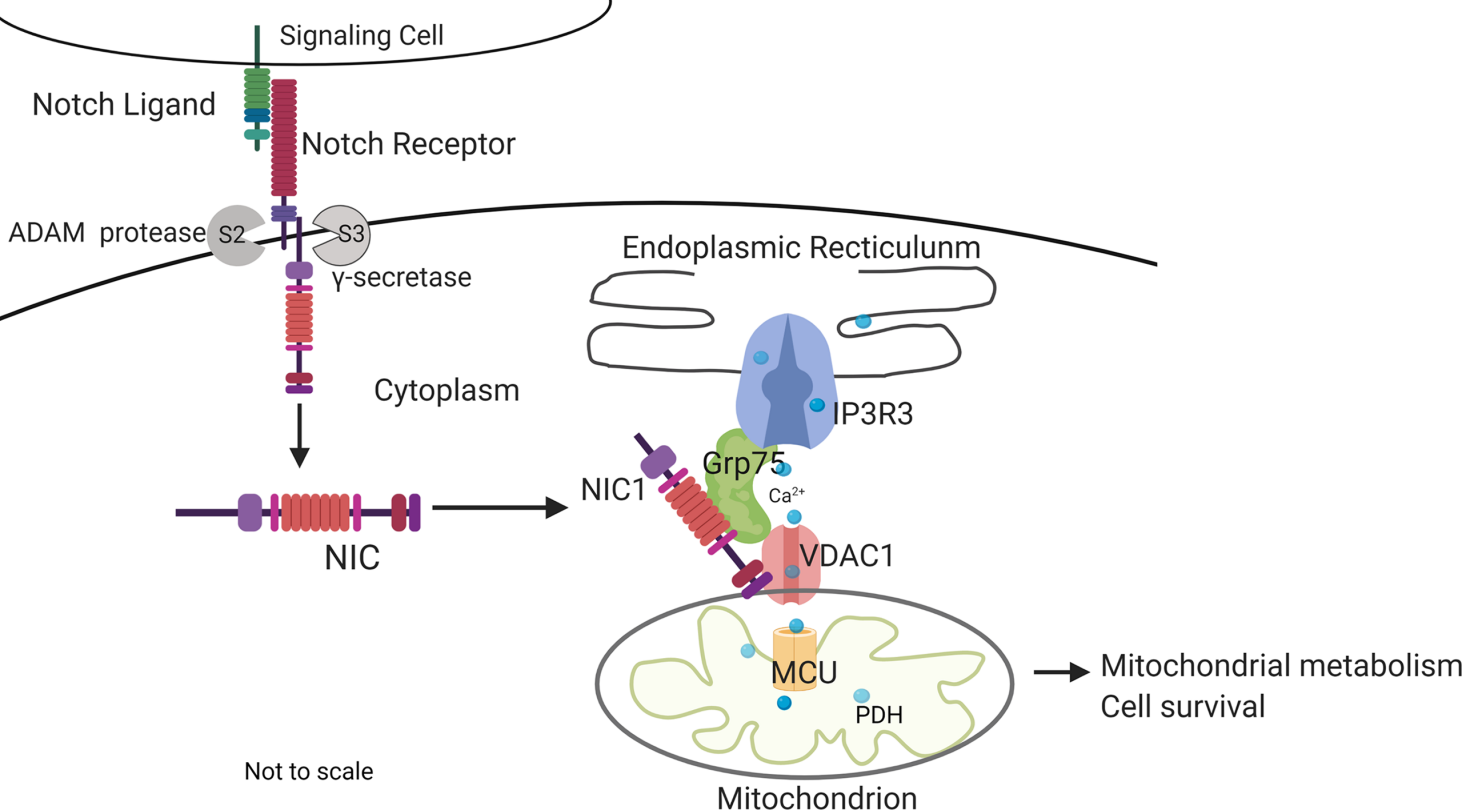
Summary of key outcomes. Non-nuclear localized NIC1 interacts with Grp75 and VDAC1 in a complex that modulates calcium homeostasis in mitochondria with consequences to cellular metabolism and survival. The schematic is not to scale.

This study builds on earlier observations of Notch1-mediated inhibition of apoptotic cascades coordinated by mitochondria (5–8) to show that Notch1 (NIC1) activity tunes calcium uptake in mitochondria, with consequences for metabolism and regulation of apoptosis in Tregs. Initial analysis in the mammalian cell line HEK showed that NIC1 activity regulated the distribution of cellular calcium as indicated by assays of calcium dynamics and direct measures of free calcium in the ER, a major store of cellular calcium. A siRNA based limited screen for molecular interactions underlying Notch-mediated outcomes revealed a dependence on IP3R3, VDAC1, Grp75, and MCU, which have well-described roles in the movement of calcium from the ER into mitochondria (31, 33, 38, 67). Building on these observations and using a combination of chemical inhibitors and functional assays in genetically ablated cells, we show that crosstalk between NIC1 and these molecules regulates mitochondrial metabolism and also confer IL-2 independent survival in Tregs (**Figure 7**).

We show that Notch1^-/-^ Tregs are characterized by deficits in mitochondrial Oxphos activity and spare respiratory reserve and present evidence to suggest that these may be linked to Notch1 regulated Grp75 protein stability. Levels of Grp75 protein are restored in Notch1 null Tregs expressing NIC-NES, with immunoprecipitation analysis providing evidence of NIC in stable association with the Grp75-VDAC1 complex in Tregs. Consistently, enforced expression of Grp75 corrected, in part, deficits in mitochondrial metabolism and conferred near complete protection from apoptosis triggered by cytokine withdrawal in Notch1^-/-^ Tregs. Further, anti-apoptotic outcomes of expressing NIC1-NES or Grp75 in Notch1^-/-^ Tregs were dependent on IP3R3 and MCU activity, linked to the regulated uptake of calcium by mitochondria. While the 2mtD3cpv probe did not detect increased levels of free calcium in mitochondria, the NIC1 mediated calcium dependent phosphorylation of PDH and Oxphos, serve as indirect indicators of continuous uptake and utilization of calcium by mitochondria (40).

Grp75 and IP3R3 are amongst the several proteins identified as constituents of dynamic molecular complexes – referred to as Mitochondria associated membranes (MAM) – bridging the ER and mitochondria (33, 34, 68). A core function of MAM is to calibrate the transfer of calcium released from the ER and its uptake into mitochondria (33, 63, 69). Perturbations of MAM are reported to reduce mitochondrial calcium uptake and increase cytosolic calcium (69, 70). The over-expression of Grp75 has also been shown to increase MAM formation and reduce resting ER calcium levels (33). In this context, a possibility arising from our studies that remain to be addressed is that Notch1 activity modulates MAM-mediated contacts. While initial efforts have not revealed measurable differences resulting from NIC1 activity in the cell line system, efforts to make reliable measurements in Tregs, which have a crowded mitochondrial organization are ongoing.

While reduced Oxphos has been shown to suppress activation of Bcl-2 family pro-apoptotic proteins Bax and Bak (71–73), the anti-apoptotic proteins Bcl-xL and Bcl2 are also reported to stimulate Oxphos and promote cell survival (74–79). Further, other evidence in the literature indicate that Oxphos promotes cellular adaptation and survival to several stresses including hypoxia, low glucose, and proteotoxic stress (80–83). An essential role for Oxphos in Treg survival in different physiological contexts including the tumor microenvironment has also been reported (84–88).

Cell survival in complex and changing environments associated with inflammation is an important component determining immune cell function. Hence, dependency on cytokine cues alone, especially for survival, may well become bottlenecks for effective function of the T-cell lineage, wherein cell-autonomous decisions of cell death and survival control immune homeostasis (89–91). Metabolic reprogramming is increasingly appreciated as a key determinant in cell fate transitions (92–96). Unsurprisingly, NIC1 activity in Tregs is revealed by IL-2 withdrawal, mimicking nutrient deprivation cues (5). Although we did not directly assess the role of ligands here, the expression of Notch ligands in activated Tregs has been reported in earlier work from our laboratory and others (5, 97). Further, ablation of the ligand Delta-like 1 is implicated in Notch1 mediated survival of activated Tregs in IL-2 depleted medium (5). We posit that while IL-2 is critical for Treg development, ligand-dependent Notch1 activity is a key adaptive response for stressors encountered in the micro-environments Tregs function in. Dependence on ligand remains an important regulatory step controlling pathway activation in these cells (5). However, Notch1 activity in Tregs may be critical in specific contexts and non-essential in others (98).

In summary, we demonstrate that cross-talk between Notch1 and components regulating cellular calcium homeostasis are important for survival and mitochondrial function in Tregs. Notch1 is implicated in instructive fate choices, including commitment to the T-cell lineage, as well as the regulation of mature T-cell function (12, 99–103). Intracellular calcium signaling is a key factor in cell signaling and physiology, controlling outcomes as diverse as proliferation, differentiation and apoptosis. Likewise, Notch signaling is a key regulator of cell fate decisions in metazoans and we speculate that the consequences to mitochondrial metabolism reported here, may be more widely prevalent and tune cell-fate decisions governed by the Notch receptor in other cell types.

## Data Availability Statement

The raw data supporting the conclusions of this article will be made available by the authors, without undue reservation.

## Ethics Statement

The animal study was reviewed and approved by Institutional Animal Ethics Committee [INS-IAE-2019/07(R1)] and Committee for the Purpose of Control and Supervision of Experiments on Animals, Government of India.

## Author Contributions

AS, NS, and SL: Conceptualization and writing. NS and SL: Experiment design, execution, analysis. PK: execution of experiments. NS: Assembly of Figures. All authors contributed to the article and approved the submitted version.

## Funding

This work was supported by extramural funding from the Department of Biotechnology DBT(BT/PR13446/COE/34/30/2015 and in part BT/PR13890/BRB/10/788/2010), as well as core support from the DBT-Institute for Stem Cell Science and Regenerative Medicine (inStem), Bellary Road, Bengaluru, India to AS.

## Conflict of Interest

The authors declare that the research was conducted in the absence of any commercial or financial relationships that could be construed as a potential conflict of interest.

## Publisher’s Note

All claims expressed in this article are solely those of the authors and do not necessarily represent those of their affiliated organizations, or those of the publisher, the editors and the reviewers. Any product that may be evaluated in this article, or claim that may be made by its manufacturer, is not guaranteed or endorsed by the publisher.

## Abbreviations

Bcl-xL, B-cell lymphoma extra-large; Foxp3, Fork head box P3; Grp75, Glucose-regulated protein 75; Hes5, hairy and enhancer of split-5; IL-2, Interleukin-2; IP3R3, Inositol 1,4,5-trisphosphate receptor, type 3; MCU, Mitochondrial calcium uniporter; NIC1, Notch1 intracellular domain; PDH, Pyruvate dehydrogenase; pPDH, Phosphorylated pyruvate dehydrogenase; RBP-jk, Recombination signal – binding protein-Jκ; Tregs, Regulatory T cells; TG, Thapsigargin; VDAC1, Voltage-dependent anion-selective channel 1.
