# Supplementary material for: Notch1 Modulation of Cellular Calcium Regulates Mitochondrial Metabolism and Anti-Apoptotic Activity in T-Regulatory Cells
Source: Front Immunol. 2022 Feb 10;13:832159. doi: 10.3389/fimmu.2022.832159 (PMC8866856; doi:10.3389/fimmu.2022.832159)
Supplement: Supplementary file 1 [file DataSheet_1.docx]

Supplementary Material

**Supplementary Figure 1**


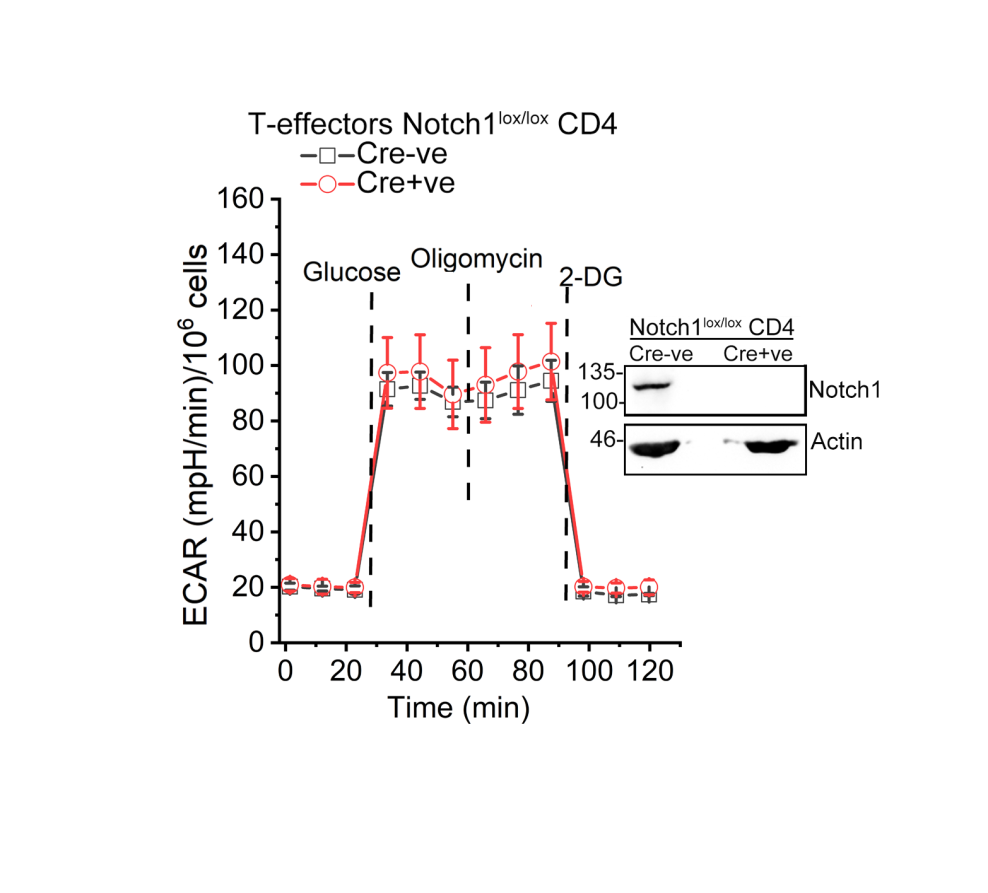


**Supplementary figure 1: ECAR in CD4^+^T-effectors.** ECAR in Notch1^+/+^ (Cre-ve; *Notch1*^lox/lox^ ()) and Notch1^-/-^ (Cre+ve; *Cd4-Cre::Notch1*^lox/lox^ ()) CD4^+^ T-effectors at baseline and in response to 10 mM glucose, 1.25 μM oligomycin and 50 mM 2-DG. Inset: Immunoblots analysis of whole cell lysates of Notch1^+/+^ and Notch1^-/-^ CD4^+^ T-effectors, for Notch1 and Actin. Data plotted are mean + SD of readings in triplicate wells and is representative of two separate experiments.


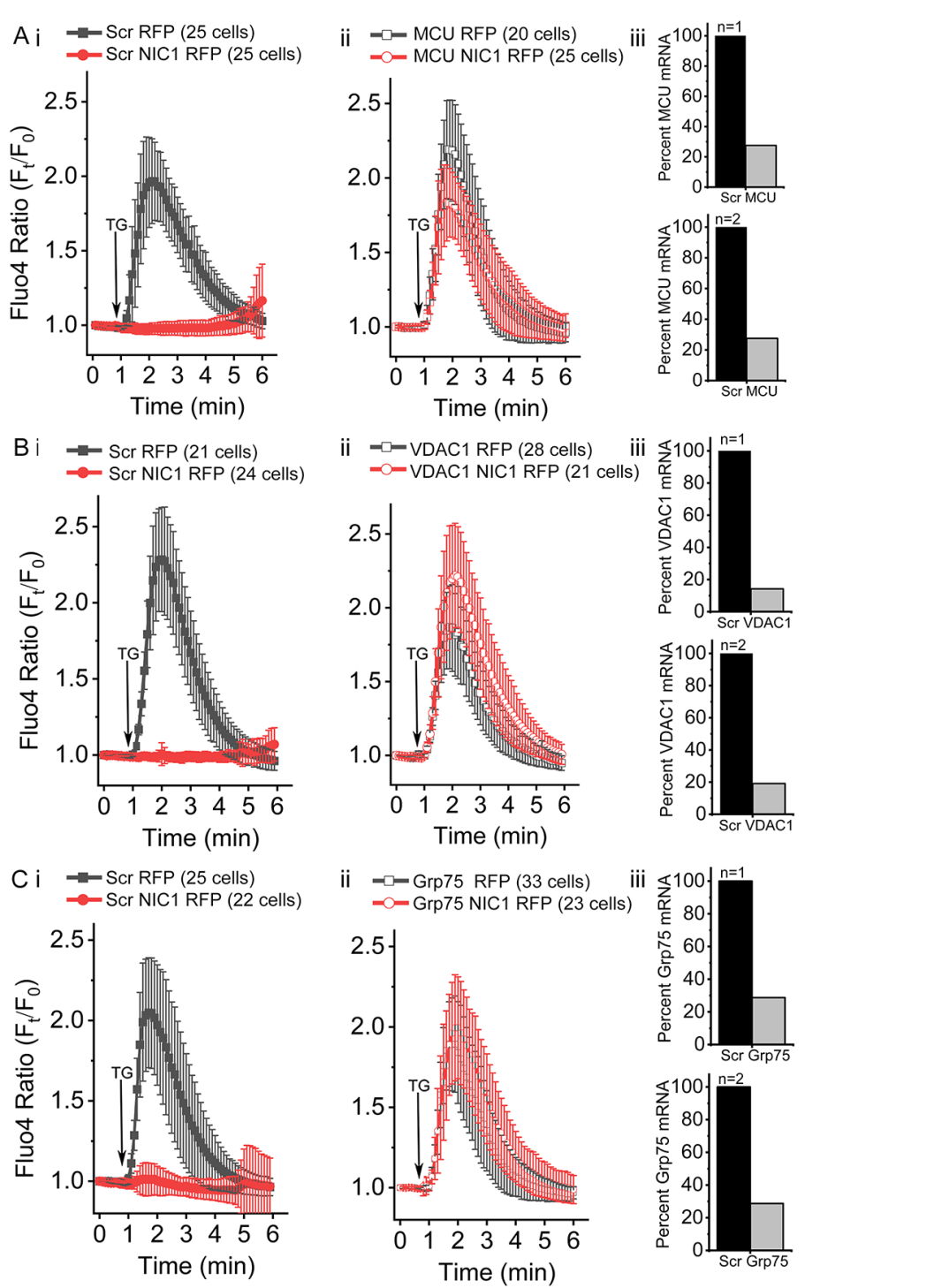
**Supplementary Figure 2**

**Supplementary figure 2: NIC1 signaling regulates ER calcium levels**. (A-C) Fluo4 fluorescence at time-t (F_t_) relative to onset (F_0_) measured in calcium free medium at baseline and in response to 2 μM TG treatment. Cells pre-treated with siRNA to MCU (A-ii), VDAC1 (B-ii) or, Grp75 (C-ii) or, scrambled control (A-C-i) for 48 h, were transfected with RFP [Scr (), siRNA ()] or NIC1 RFP [(Scr (), siRNA ()] and cultured for 24 h in complete medium, before analysis in the passive store depletion assay as described in methods. (A-C iii) Percent mRNA of the genes as shown in panels, in cells treated with MCU (A-iii), VDAC1 (B-iii) or Grp75 (C-iii) and scrambled control. Data plotted as mean+SD of the indicated number of cells across two independent experiments.

**Supplementary Figure 3**

**
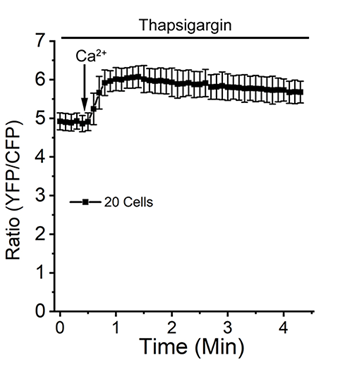
**

**Supplementary figure 3: 2mtD3cpv detects change in mitochondrial calcium levels**. YFP/CFP fluorescence ratio in HEK cells expressing 2mtD3cpv, at baseline and in response to 2 mM calcium addition following the treatment with 2 µM TG. Data plotted as mean+SD of the indicated number of cells across two independent experiments.

**Supplementary Figure 4**

**
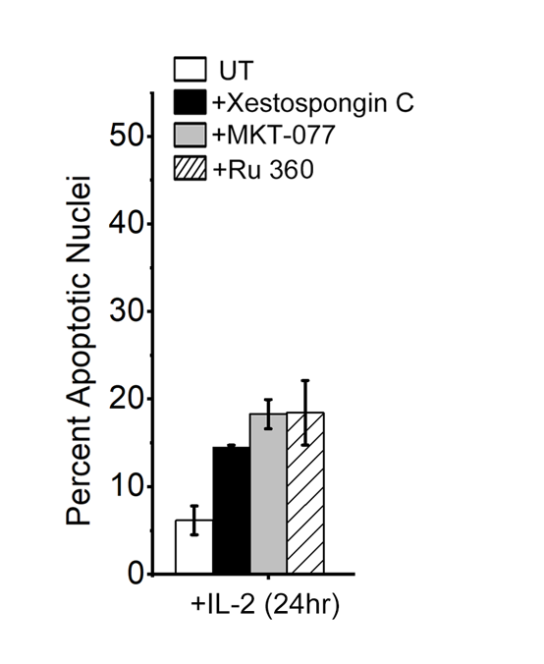
**

**Supplementary figure 4: IP3R3, Grp75 and MCU are not required for Tregs survival in presence of IL-2**. Percent apoptotic nuclei in activated Tregs cultured with IL-2 (1 µg/ml) for 24 h with or without 5 μM Xestospongin C or, 10 µM MKT-077 or, 10 μM Ru360. Data represent mean+SD of three independent experiments**.**

**Supplementary Figure 5**

**
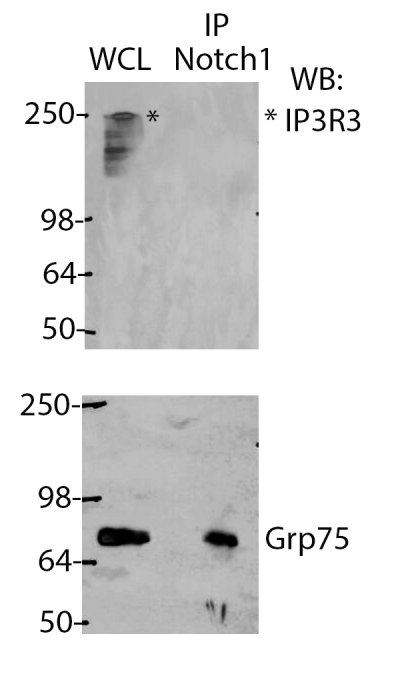
**

**Supplementary figure 5: Notch1 does not interact with IP3R3.** Cell lysates of activated WT Tregs were subjected to immunoprecipitation using an antibody to Notch1 and associated proteins analyzed by western blotting for IP3R3 (*shows IP3R3 band) and Grp75. Immunoblot is representative of two independent experiments.
